# The effect of a single consecutive volume aspiration on concentrated bone marrow from the proximal humerus for clinical application

**DOI:** 10.1186/s12891-019-2924-2

**Published:** 2019-11-14

**Authors:** Lukas N. Muench, Cameron Kia, Alexander Otto, Julian Mehl, Joshua B. Baldino, Mark P. Cote, Mary Beth McCarthy, Knut Beitzel, Augustus D. Mazzocca

**Affiliations:** 1 Department of Orthopaedic Sports Medicine, Technical University, Munich, Germany; 2 Department of Orthopaedic Surgery, University of Connecticut, Farmington, CT USA; 3 Department of Shoulder Surgery, ATOS Clinic, Cologne, Germany

**Keywords:** Bone marrow aspiration, Proximal humerus, Shoulder surgery, Biologic augmentation

## Abstract

**Background:**

Low aspiration volumes have been recommended to allow for higher concentrations of progenitor cells during bone marrow harvesting. However, these guidelines then require multiple aspiration attempts in order to maximize cellular yield. The purpose of this study was to investigate the effect of a single, high-volume aspiration with four consecutive aliquots on the number of nucleated cells (NCs) and colony-forming units (CFUs) in concentrated bone marrow aspirate (cBMA) taken from the proximal humerus.

**Methods:**

cBMA was taken from the proximal humerus of patients undergoing arthroscopic rotator cuff surgery. Four 12-mL double syringes were used consecutively from a single trocar to obtain four 10 cc aliquots. Each then underwent centrifugation to create a fractionated layer rich in nucleated cells. Following cellular separation, NCs were counted and CFUs were evaluated after incubation of 7–10 days. Cellular comparisons between each aliquot were performed along with their interaction with patient age and sex.

**Results:**

Twenty-nine patients (55.9 ± 4.6 years) were included in this study. The number of NCs and CFUs showed significant differences between the four aliquots of aspirate, with the first 10 cc aliquot providing the highest amount (*p* < 0.001, respectively). No significant differences were found between the sum of the three sequential aliquots compared to the initial 10 cc sample. There were no significant differences between male and female patients (*p* > 0.05). Increasing age resulted in no significant decrease in the number of NCs and CFUs across the four consecutive aliquots (*p* > 0.05).

**Conclusion:**

In conclusion, while the initial aliquot provided the greatest number of nucleated cells and cultured CFUs, the addition of each sequential volume aspirate yielded similar amounts in total. This demonstrates the potential effectiveness of obtaining of higher volume aspirates from the proximal humerus during rotator cuff repair.

## Background

Biologic cell-based therapy has garnered increasing attention over the past two decades [1–3], with recent research attempting to optimize harvest and processing methods to increase the number of pluripotent stem cells [4, 5]. In the field of orthopaedics, various sources have been examined in order to obtain these progenitor cells, however, bone marrow aspirate is the current gold standard [6].

Although various locations may be used to obtain bone marrow, the quality and effectiveness of an aspirate still depends on the concentration of connective tissue progenitors (CTPs) within the sample [7]. According to Muschler et al., this concentration is influenced by the volume of aspirate [8]. The authors found that smaller samples may contain a higher concentration due to larger volumes becoming diluted with peripheral blood [8]. The authors compared one-milliliter, two-milliliter and four-milliliter aspirates obtained from the anterior iliac crest [8]. They found that using one-milliliter aspirates four times would provide almost twice the amount of colony forming units (CFUs) compared to one four-milliliter aspirate [8]. Therefore, aspiration techniques using multiple aspirations of smaller volumes in order to limit dilution with peripheral blood would ensure a higher concentration of CTPs [5, 8, 9].

Preventing dilution of a bone marrow aspirate is of clinical concern, as effectiveness of bone marrow aspirate (BMA) applications depends on the total number of CTPs in the sample [10]. With the percentage of mesenchymal stem cells comprising as little as 0.01% of a bone marrow aspirate, the process of centrifugation to increase the nucleated cell count has become of clinical importance [11]. Using a centrifuge, cellular components of the sample can be separated, allowing for generating a highly concentrated BMA (cBMA) sample regardless of prior dilution with peripheral blood. Mazzocca et al. first described an arthroscopic technique for obtaining bone marrow aspirate from the proximal humerus [4]. This technique allows the harvest site to later be used for placement of the medial anchor during rotator cuff surgery, thus decreasing donor site morbidity [4]. Using this method, a previous study by Voss et al. found that CTPs could be present even after 40 ml of bone marrow aspiration from the same harvest site [12]. However, their study was limited in its focus on aspiration technique, and did not evaluate patient factors or the sequential decrease in CFUs with each aliquot obtained [12].

The purpose of the current study was to investigate the effect of four consecutive aliquots from a single high-volume aspiration on the number of nucleated cells and CFUs in concentrated bone marrow aspirate taken from the proximal humerus. Age and sex were evaluated as secondary outcome measures. The authors hypothesized that there would be continued presence of nucleated cells and CFUs even after the fourth aliquot, with no significant differences between the first and second 10 cc aliquot. The secondary hypothesis was that increasing age would result in a significant decrease in the number of nucleated cells and CFUs across the four aliquots.

## Methods

### Patient selection

Patients undergoing primary rotator cuff surgery between December 2010 and June 2014 were considered for enrollment in the study. Institutional review board approval was obtained prior to the initiation of the study (IRB No. 06–577-2). Nine of the 29 patients included in this study were also enrolled in the study by Voss et al. comparing trocar fenestration [12]. All nine of these patients underwent non-fenestrated trocar aspiration [12]. Patients were eligible for inclusion if they were 18 years or older and were indicated for primary arthroscopic rotator cuff repair after failing conservative treatment. All samples of bone marrow aspirate were obtained from the proximal humerus by a fellowship trained shoulder surgeon (ADM), using a previously described method [4].

Patients in “vulnerable populations”, such as pregnant women and prisoners were excluded from the study. Exclusion criteria also included any history of a systemic infectious disease (e.g., hepatitis, human immunodeficiency virus, etc.) or bone marrow derived illness (e.g., leukemia) as well as previous exposure to therapeutic radiation (e.g., cancer treatment). Patients were also excluded if they had prior stem cell injections.

### Bone marrow aspiration

**Fig. 1:**
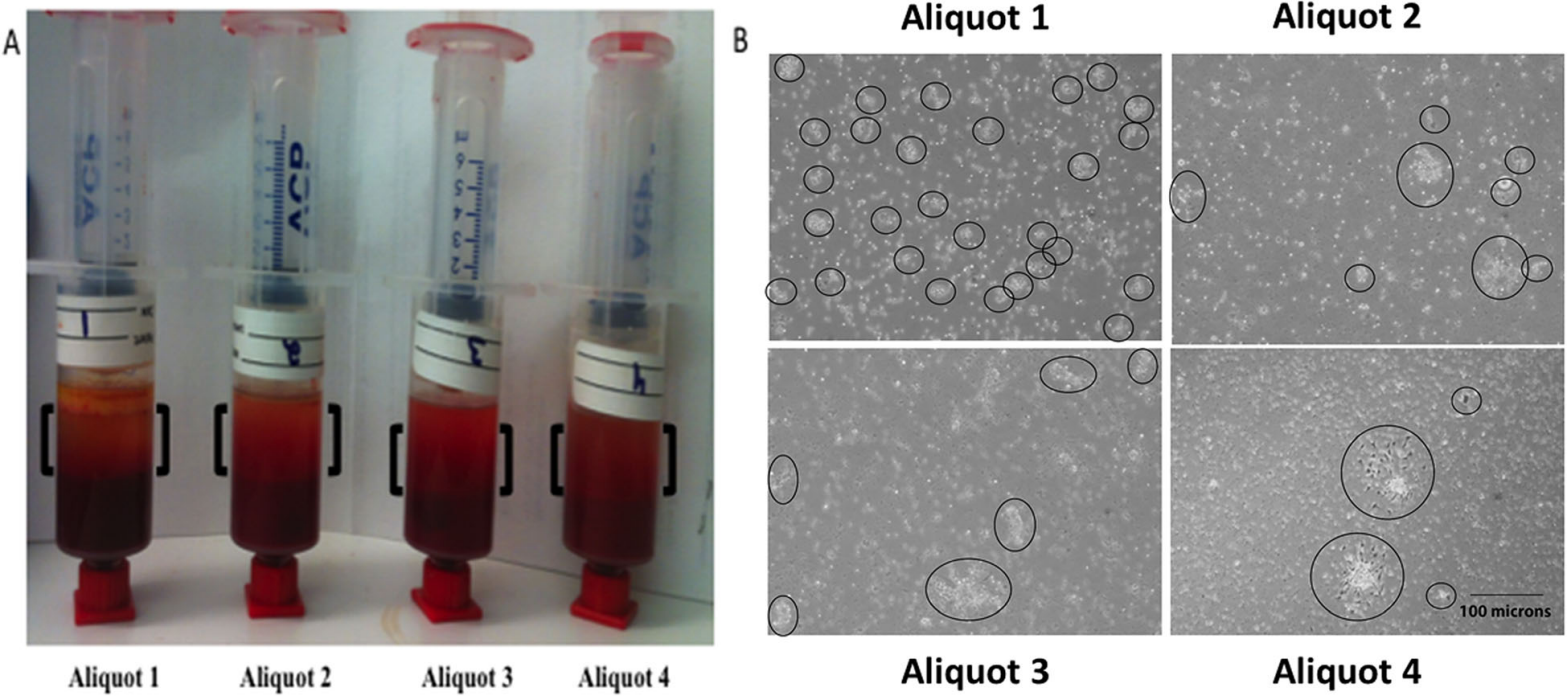
Demonstrating the fractionated layer (black parenthesis) of each of the four consecutive aliquots following centrifugation (**a**). **b** demonstrates an example of the colonies formed (circled) comparing the different aliquots

Bone marrow was aspirated from the proximal humerus during arthroscopic rotator cuff surgery according to a previously published method [4]. Prior to aspiration, a non-fenestrated trocar (11-gauge) (Arthrex, Naples, FL, USA) was flushed 3 times with a heparin/saline solution containing 1000 IU/ml. Four 12-ml double syringes (aliquots 0–10 cc, 11–20 cc, 21–30 cc, 31–40 cc) (Arthrex, Naples, FL, USA) were used for each consecutive aspiration of 10 cc of bone marrow (Fig. 1). Prior to aspiration, 2 cc of 1000 IU/ml heparine solution were added to each syringe.

First, the 11-gauge trocar was fit with a 12-ml double syringe and inserted 2.5 to 3 cm into the medial aspect of the greater tuberosity (Bone Marrow Aspiration Kit, Arthrex, Naples, FL, USA). The surgeon then pulled on the syringe plunger until each syringe was filled to the maximum volume of bone marrow available (goal of 10 cc/syringe). Keeping the depth of the trocar constant, the trocar was turned 90° after each volume of aspiration was collected.

### Bone marrow processing and evaluation

As previously described [12], the harvested BMA was spun in the four double syringes using a centrifuge (Roto fix 32 A, Hettich, Tuttlingen, Germany) at 800 rpm for 5 min to obtain a fractionated layer. After spinning, the top fractionated layer containing the nucleated cells and the concentrated bone marrow stromal cells (cBMA) was drawn into the inner syringe, being careful not to include the erythrocyte layer [12]. This resulted in ~ 3–7 cc of product rich in nucleated and concentrated bone marrow stromal cells, which was brought to the laboratory for cellular counting using the Z1 Coulter Counter (Beckman Coulter Life Sciences, Indianapolis, IN, USA). In order to obtain the total nucleated cell count, 0.1 cc of cBMA from each of the four syringes was diluted in 9.9 cc of saline. The number of nucleated cells in this 10 cc of solution was calculated and multiplied by 10 to obtain the total number of nucleated cells in 1 cc of cBMA.

One milliliter of cBMA from each of the four syringes was plated in 100 mm Primaria culture dishes (Fischer Scientific, Pittsburgh, PA, USA) with 9 cc of phenol red-free alpha-minimum essential media (MEM) containing 10% fetal bovine serum (FBS) (Thermo Fisher Scientific, Waltham, MA, USA) and 0.1% penicillin streptomycin sulfate (Thermo Fisher Scientific, Waltham, MA, USA). Cells were placed in 37 °C with 5% CO_2_. Media was changed after 24 h to remove the non-adherent cells and plates were checked daily for the appearance of colonies and contamination. Colony forming units were counted after 7–10 days under the microscope (Eclipse TS100, Nikon Instruments Inc., NY, USA) by the same experienced investigator each time [12]. A colony was defined as a cluster of 8 or more cells [13]. The CFUs were counted for one quarter of the plate and multiplied by four to obtain the total number of CFUs per 1 cc of cBMA.

### Statistical analysis

Descriptive statistics are reported as mean and standard deviation (+/−). To account for the non-normal distribution of the cell data, two separate mixed-effects generalized linear models using the Poisson distribution (log link) were fit to examine the effects of the four consecutive aliquots on NC count and number of CFUs. Pairwise comparison of mean values was carried out to determine a difference between each aliquot of bone marrow aspirate and sex. The interaction between the aliquot of aspirate and age was added to the model to determine whether increasing age had a differential effect on the mean number of nucleated cells and colony-forming units across the four aliquots of bone marrow aspirate. Results of all inferential analyses are reported as mean difference with corresponding 95% confidence intervals. To account for multiple comparisons, *p*-values were adjusted according to the Bonferroni’s method. A *p* < 0.05 was determined to be statistically significant. All statistical analyses were performed using Stata (StataCorp 2017. Stata Statistical Software: Release 15. College Station, TX: StataCorp LLC).

## Results

### Volume of BMA and cBMA

Twenty-nine patients were included in this study with a mean age of 55.85 ± 4.58 years (9 females and 20 males). Ten milliliters of bone marrow was successfully aspirated into each of the four syringes, resulting in an average volume of 40.0 ± 0.0 mL of BMA per patient. After 5 min of centrifugation, the average volume of the concentrated layer of BMA showed no significant difference between the four consecutive aliquots of aspirate (Fig. 1a). The first aliquot (0–10 cc) resulted in an average concentrated volume of 3.3 ± 1.2 cc, the second aliquot (11–20 cc) was 3.1 ± 1.0 cc, the third aliquot (21–30 cc) was 3.2 ± 0.9 cc, and the fourth aliquot (31–40 cc) resulted in a volume of 3.3 ± 1.2 cc (*p* > 0.05, respectively).

### Nucleated cell count

**Fig. 2:**
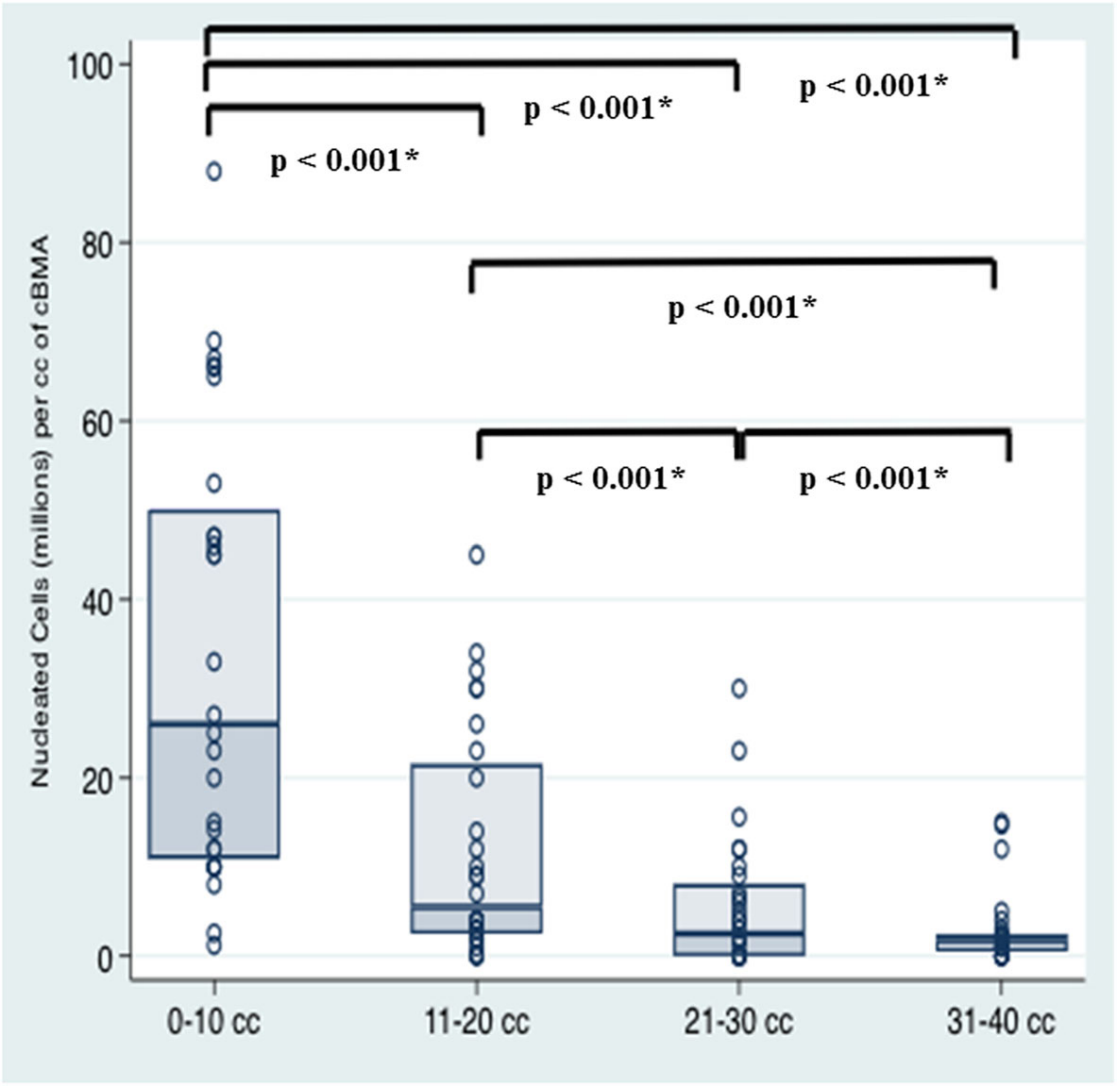
Demonstrating the number of nucleated cells (millions) per cc of cBMA stratified by the four aliquots (0–10 cc, 11–20 cc, 21–30 cc, 31–40 cc) of bone marrow aspirate. * Indicates statistical significance

There were significant differences found in nucleated cell count between the four aliquots of bone marrow aspirate (Fig. 2). With a mean of 30.7 ± 23.5 × 10^6^, the first 10 cc of aspirate provided the highest number of nucleated cells per cc of cBMA (median, 24.0 × 10^6^ nucleated cells), whereas the second aliquot (11–20 cc) averaged 13.1 ± 13.7 × 10^6^ nucleated cells per cc of cBMA (median, 8.0 × 10^6^) (*p* < 0.001). The third (21–30 cc) aliquot (mean: 6.8 ± 9.6 × 10^6^; median: 3.5 × 10^6^) had significantly less nucleated cells when compared to the first and second aliquot (p < 0.001, respectively), whereas the fourth (31–40 cc) aliquot (mean: 3.0 ± 4.3 × 10^6^; median: 2.0 × 10^6^) had significantly less nucleated cells than all the other aliquots (p < 0.001, respectively). When comparing the mean number of NCs in the first aliquot to the sum of the averages of the second, third, and fourth aliquot, there was no significant difference (*p* > 0.05). There was no significant difference in the mean number of NCs of each consecutive aliquot when compared between male and female patients (p > 0.05).

### Colony-forming units

**Fig. 3:**
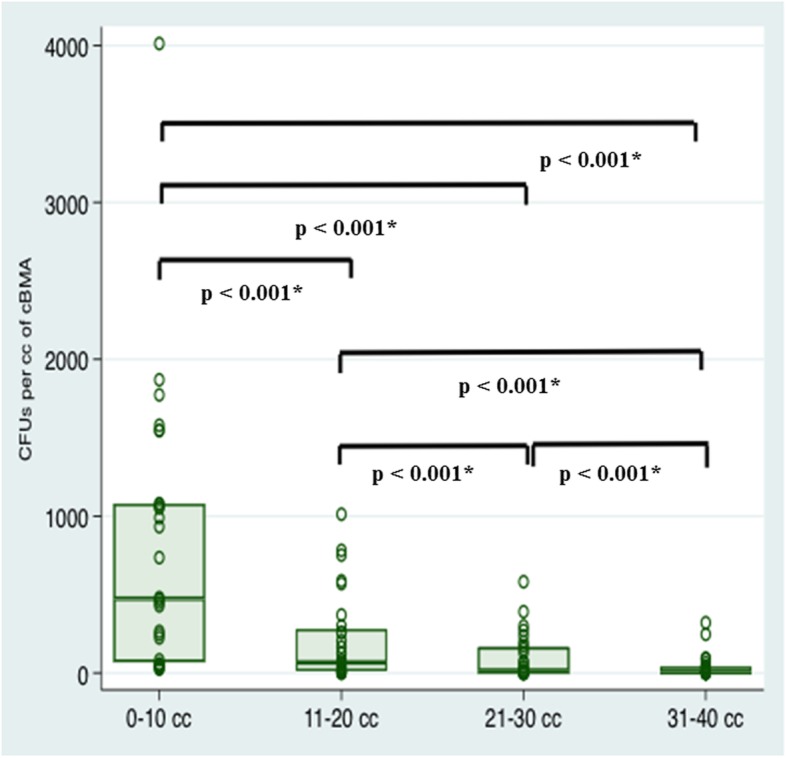
Demonstrating the number of CFUs per cc of cBMA stratified by the four aliquots (0–10 cc, 11–20 cc, 21–30 cc, 31–40 cc) of bone marrow aspirate. * Indicates statistical significance

Colony-forming units (CFUs) were evaluated after 7–10 days of cell culture as an estimated measurement for the prevalence of CTPs (Fig. 1b). The number of CFUs showed a significant difference between the four aliquots of bone marrow aspirate (Fig. 3). For the first 10 cc of aspirate, a mean of 742.0 ± 885.9 CFUs (median, 458.0) were counted per cc of cBMA. The second aliquot averaged 227.3 ± 296.7 CFUs (median, 78.4), whereas the third (mean: 123.5 ± 199.2; median: 27.4) and fourth (mean: 39.3 ± 77.9; median: 3.1) aliquot provided the least numbers of CFUs per cc of cBMA (*p* < 0.001, respectively). When comparing the mean number of CFUs in the first aliquot to the sum of the averages of the second, third, and fourth aliquot, there was no significant difference (*p* > 0.05). There was no significant difference in the mean number of CFUs of each consecutive aliquot when compared between male and female patients (p > 0.05).

### Influence of age on number of nucleated cells and Colony-forming units

**Fig. 4:**
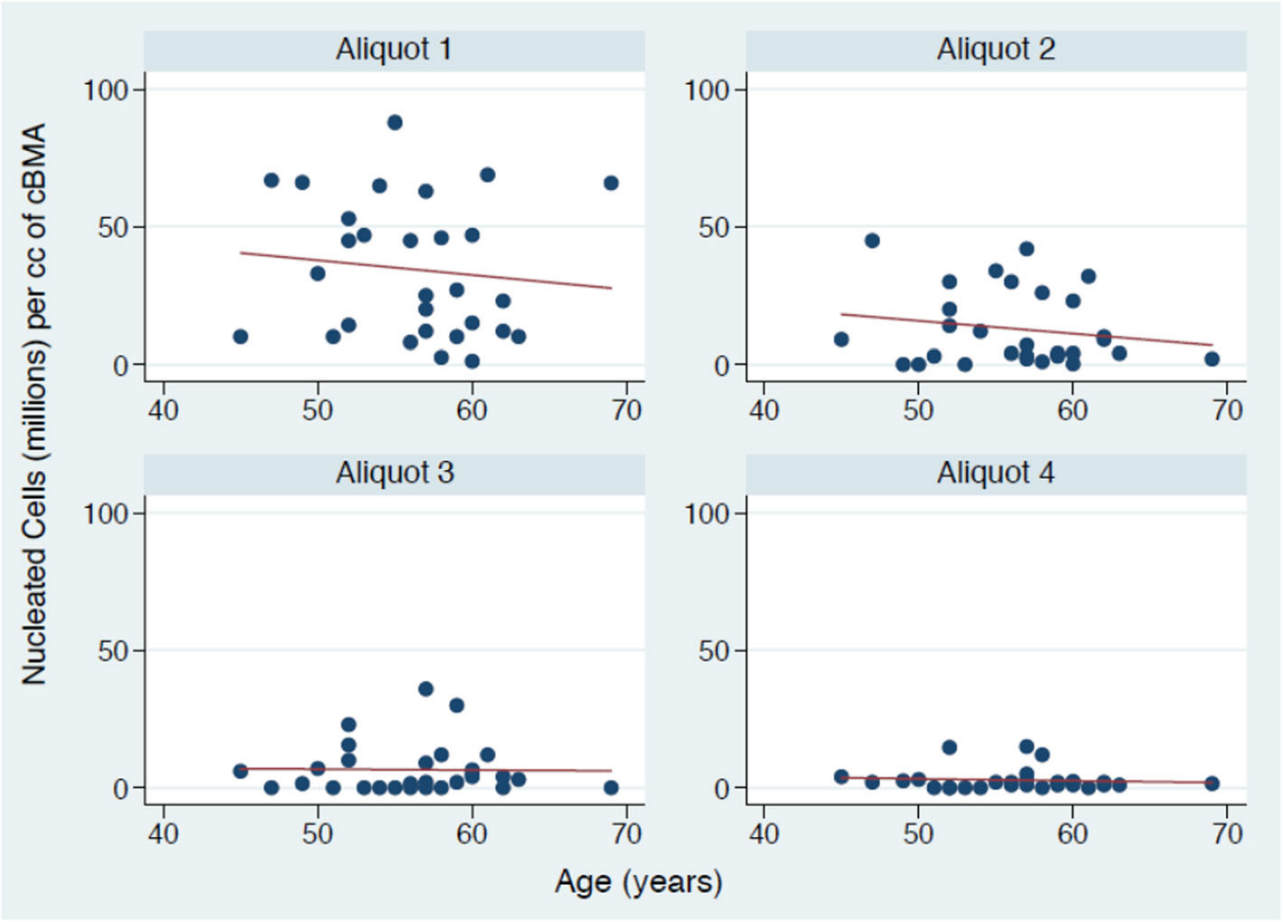
Demonstrating the aliquot of aspirate by age interaction influencing the number of nucleated cells (millions) per cc of cBMA

**Fig. 5:**
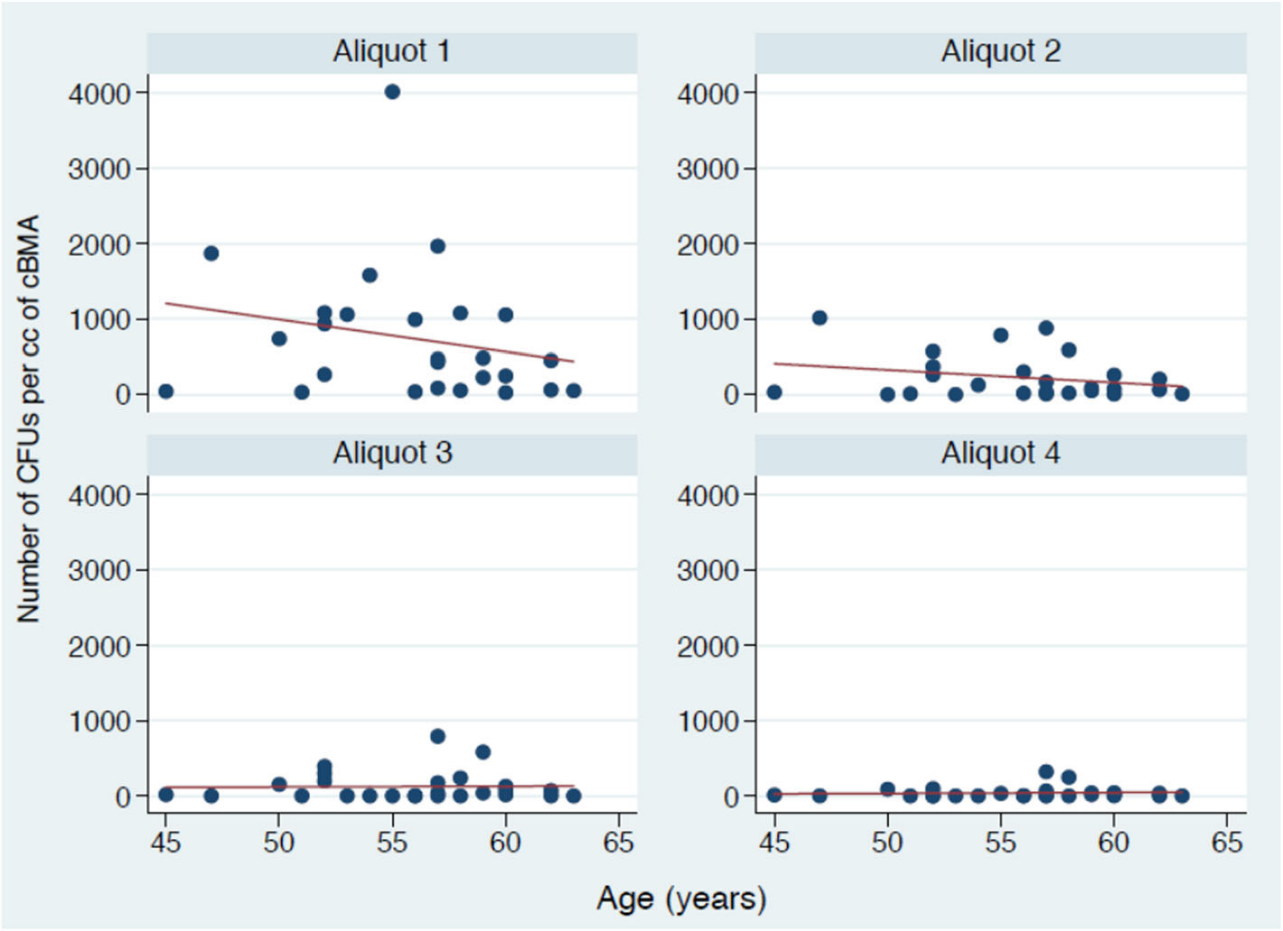
Demonstrating the aliquot of aspirate by age interaction influencing the number of CFUs per cc of cBMA

The interaction between age and consecutive aliquot of aspirate was then examined. This interaction demonstrated that increasing age had no significant effect on the mean number of NCs across the four aliquots of bone marrow aspirate (Fig. 4) (p > 0.05, respectively). Similarly, increasing age had a no differential effect on the mean number of CFUs across the four aliquots of bone marrow aspirate (p > 0.05,respectively) (Fig. 5).

## Discussion

The most important finding of this study is that despite significantly less nucleated cells and CFUs per sequential aliquot, connective tissue progenitor cells were still found to be present after aspiration of 40 ml of bone marrow from the proximal humerus. In addition, when comparing the mean number of NCs or CFUs in the first aliquot to the sum of the averages of the second, third, and fourth aliquot, there was no significant difference. Increasing age of the patient, resulted in no significant decrease in the number of NCs and CFUs across the four consecutive aliquots.

In order to avoid hemodilution, several studies have supported the use of repeated smaller aspirates, ranging from 1 to 4 ml [8, 14, 15]. However, multiple small aspirations form a single trocar or from various sites may lead to longer surgical time, risk of infection or damage to nearby neurovascular structures [16]. Hernigou et al. examined the effect of syringe size on mensenchymal stem cell (MSC) aspiration, and found that aspirating only part of the tube’s full capacity (10–20%) produced a higher MSC concentration than aspirating either a 10 mL or 50 mL tube to completion [17]. However, their study focused on single aspiration from multiple sites of the iliac crest, rather than the cumulative effects of fractioning samples from a single aspirate [17].

Voss et al. found that using a non-fenestrated trocar yielded significantly higher cell counts, which was the technique performed in this study [12]. While they demonstrated continued prevalence of CTPs with sequential aspiration, they were limited in number of patients as well as the influence of fractionated layer volume following centrifugation [12]. We found that each aliquot had similar amounts of hemodilution with no effect on the final number of nucleated cells and CFUs in the sample. In addition, we demonstrated that the sum of CFUs from the sequential aliquots yielded similar progenitor cells compared to the initial 10 cc of aspiration. This is of clinical importance, as efficacy of mesenchymal stem cells has direct correlation with the quantity of these cells [18]. This study also highlights the importance of age on the number of CFUs and nucleated cells within the first fractioned aliquot, as well as its impact on subsequent concentrated aliquots. This supports previous findings that the number of osteoprogenitor cells in bone marrow demonstrates marked decrease after the first two decades [19].

The efficacy of autologous bone marrow for clinical applications depends on the concentration of necessary progenitor cells [10]. While certain patient characteristics, such as alcohol abuse [20] and smoking [21] can negatively affect bone marrow aspirate quality, optimizing surgical technique is essential for success of treatment. Although aspiration of bone marrow from the iliac crest is still considered the gold standard [5, 22–24], complications such as hematoma and nerve palsy have been reported [25]. While the proximity of the axillary nerve and artery make the proximal humerus amenable to similar risks, the ability to obtain the sample under direct visualization during rotator cuff repair makes this an ideal location. Mazzocca et al. first described the proximal humerus as a more desirable source of MSCs for rotator cuff repair due to its ease of attainment [4]. Along with attainment of these cells, quantifying their type and characteristics has varied in the literature. The International Society of Cell Therapy defined adult stem cells that adhere to culture plastic and express markers such as CD73, CD90, and CD105 [13]. Juneja et al. demonstrated a viable method for aspiration of mesenchymal stem cells from the femur of patients undergoing total hip and knee arthroplasty [26]. Using flow cytometry, the authors demonstrated that cells obtained were positive for several markers (C73, CD105, CD90), meeting the criteria for stem cell criteria [26]. In this study, colony-forming units was used as a measurement for the number of connective tissue progenitor cells. As analysis of CFUs may not be ideal intraoperatively, El-Jawhari recently proposed a novel rapid flow cytometry assay that can be used to quantify bone marrow-derived MSCs within an intraoperative time frame [27].

There are several limitations to this study. This is an in-vitro study without direct evidence of the effects of these implanted cells. In addition, nucleated cell count and the CFU assay used in this study do not give information about the heterogeneity of the CTPs and colonies they form [28]. Another limitation is the wide inter-individual variability with regards to counting the number of nucleated cells and CFUs, making comparisons to prior studies difficult [8, 22, 26, 29]. This includes disparities as to when a CFU should be counted (e.g., 7, 10, and 14 days), as well as what should be the minimum number of aggregated cells used to define a CFU, which has been shown to have gross variability (e.g., 8 and 50) [26, 30, 31]. Accordingly, counting small colonies may generate higher total numbers compared to studies in which a colony was defined as a cluster of significantly more cells, which may lead to misinterpretation of the data. In addition, these methods are dependent on the expertise in microscopic evaluation of those examining [26, 30]. In this study, an experienced research associate (MBM) performed the colony counting and a CFU was defined to be a cluster of 8 or more cells [13]. However, counting CFUs may underestimate the actual number of CTPs as more than one CTP can give rise to a colony [28]. Additionally, this study had no comparison group to evaluate if the findings are consistent with aspiration of other sites, such as the iliac crest or vertebral body.

## Conclusion

In conclusion, while the initial aliquot provided the greatest number of nucleated cells and cultured CFUs, the addition of each sequential volume aspirate yielded similar amounts in total. This demonstrates the potential effectiveness of obtaining of higher volume aspirates from the proximal humerus during rotator cuff repair.

## Data Availability

The datasets generated and analyzed during the current study are not publicly available due, but are available as de-identified data sheet from the corresponding author on reasonable request.

## Abbreviations

BMA: bone marrow aspirate
cBMA: concentrated bone marrow aspirate
CFU: colony-forming unit
CTPs: connective tissue progenitors
FBS: fetal bovine serum
MEM: minimum essential media
MSC: mesenchymal stem cell
